# Polymerase Chain Reaction and Its Application in the Diagnosis of Infectious Keratitis

**Published:** 2019

**Authors:** Harry Y. Liu, Grant C. Hopping, Uma Vaidyanathan, Yasmyne C. Ronquillo, Phillip C. Hoopes, Majid Moshirfar

**Affiliations:** 1 Health Science Center, McGovern Medical School, University of Texas, Houston, TX, USA; 2 John A. Moran Eye Center, Department of Ophthalmology and Visual Sciences, School of Medicine, University of Utah, Salt Lake City, UT, USA; 3 Utah Lions Eye Bank, Murray, UT, USA; 4 Hoopes Durrie Rivera Research Center, Hoopes Vision, Draper, UT, USA

**Keywords:** Polymerase Chain Reaction, Staining and Labeling, Culture, Keratitis, Fungi, Virology

## Abstract

PCR involves a repeating cycle of replication to amplify small segments of deoxyribonucleic acid (DNA). A novel application of this technique is microbial identification in infectious keratitis, one of the leading causes of blindness in the world. PCR is more sensitive than biological stains and culture, which are considered the current gold standards for diagnosing infectious keratitis. The diagnosis and treatment of infectious keratitis cost the United States millions of dollars in health expenditure. PCR may help offset that cost by allowing for individualized disease management and screening for multiple antibiotic-resistant genes. While beneficial, PCR demonstrates lower specificity rates compared to culture and stain, indicating its shortcomings; this can be overcome by performing PCR after narrowing the pool of potential microorganisms. This article examines the clinical utility of PCR in cases of infectious keratitis by evaluating its reliability, validity, associated costs, and indications.

## INTRODUCTION

Infectious keratitis is characterized by inflammation of the cornea due to infection by ﻿bacteria, fungi, amebae, and viruses [1]. It is imperative to diagnose and treat these infections as soon as possible due to the potential for vision loss. Corneal opacification, which is a sequela of infectious keratitis, is the fourth leading cause of blindness in the world [2, 3]. Trauma with subsequent microbial infiltration of the cornea accounts for up to 5% of all cases of blindness in the developing world [4, 5]. Currently, the gold standard diagnostic tests are Gram stain and culture [2, 4]. Microbial culture, in particular, has high specificity but is a relatively insensitive method [6]. However, the advent of the polymerase chain reaction (PCR) has led to multiple reports showing superior sensitivity compared to traditional methods. Commonly used staining and culturing media include the Giemsa stain, Gram stain, blood agar, and Sabouraud agar, although others are available depending on the targeted microorganism [4]. Culturing can take several days to show a diagnosis; specifically, bacterial cultures can take 2-4 days, and fungal cultures can take 2-10 days [7]. Traditional viral cultures can take days to weeks depending on the virus, although more modern culturing methods can decrease the time to 24 hours [8]. On the other hand, staining of corneal scrapings can provide immediate identification of the causative agent [2]. 

The purpose of this article is to examine the practical applications, diagnostic validity, and cost-effectiveness of PCR in infectious keratitis.

## METHODS

To find information on PCR and infectious keratitis, a literature search was performed using the following sources: PubMed, Google Scholar, and Scopus with the keywords “PCR and infectious keratitis,” “polymerase chain reaction keratitis,” “PCR and bacterial keratitis,” “PCR and fungal keratitis,” “PCR and viral keratitis,” and “PCR and keratitis.” Reference lists of these articles were used to find additional articles. There were no language restrictions. Publications were drawn between the dates of 1990-2019.

**PCR Advantages**

PCR involves repeated cycles of denaturation, amplification, and replication, in which segments of deoxyribonucleic acid (DNA) are continuously multiplied. Specific DNA primers are employed to indicate the presence of the microorganism in question [9]. PCR has also been used throughout the field of molecular biology, helping researchers clone and sequence genes for the detection of mutations [10]. It has more recently become a modality for detecting microbial agents, requiring only a small sample for analysis [7, 9]. Currently, PCR is just a confirmatory test with its use limited to the diagnosis of herpetic keratitis [4]. The PCR procedure takes approximately 4-8 hours, which is about three times faster than those of cultures [7, 11]. A particular type, known as multiplex PCR, can amplify multiple sequences of DNA in one reaction. It has been used to detect several different antibiotic genes found in methicillin-resistant staph aureus (MRSA) [12, 13], earning the distinction as the gold standard for the detection of MRSA [14].

Multiple reports have shown PCR to be comparable, if not better than, traditional staining and culturing [4, 15-17]. Gram staining can correctly identify bacteria and fungi 60-75% and 35-90% of the time, respectively [2, 18]. Cultures have a similar result, identifying 59% of bacteria and 45% of fungi [18]. The sensitivities produced by these tests are relatively low and show considerable variation, leaving room for diagnostic improvement. Polymerase chain reaction demonstrated higher positivity rates and sensitivity than culture and stains for both bacteria and fungi [7, 15]. Zhao et al. reported the positive detection rate of PCR as 83% and 85% for bacterial and fungal keratitis, respectively. For fungal keratitis, this rate was greater than the positive detection rate for culture (35%) and stain (65%) [15]. Overall, studies have reported PCR having a higher sensitivity for infectious keratitis compared to culture (98% versus 47%), but a slightly lower specificity (83% versus 100%) [15]. In regards to Acanthamoeba keratitis, PCR displayed higher sensitivity (33% vs. 71%) and comparable specificity (100%) to culture [16]. In summary, PCR appears to show superior sensitivities but comparable specificities for the identification of bacteria, fungi, and Acanthamoeba in infectious keratitis (Table 1).

**PCR Disadvantages**

PCR has a few shortcomings (Table 1). Its specificity is potentially lower than culturing and staining, implying an increased risk for false positives. Since specific primers are used to identify different microorganisms, physicians often need to list potential microorganisms before performing selective PCR [17]. For example, 16S bacterial DNA primers and 18S fungal DNA primers are the most common primers selected for these causes of infectious keratitis. Due to genetic similarities between different bacteria and fungi, common DNA may be amplified leading to false positives by the detection of normal flora of the corneal external environment [4]. Kim et al. reported another problem when they discovered organisms unrelated to human infection in their control PCR samples. They attributed this finding to airborne contamination due to their samples being shipped from India [6]. However, airborne contamination is an infrequent occurrence in PCR, implying that either this was an isolated finding or that keratitis samples are more likely to be contaminated [19]. Cross contamination is also an issue in culture and staining, but perhaps the sensitive nature of PCR increases that risk. Moreover, different microorganisms populate distinct geographical areas [20]. In a ten year study, Ting et al. found that MRSA-related keratitis was much lower in their region compared to other countries, suggesting that clinicians take locality into account when requesting and interpreting PCR results [20].

**Table 1:** Advantages and Disadvantages for the Use of Polymerase Chain Reaction (PCR) in Identifying Causative Agents in Infectious Keratitis

Advantages of PCR	Disadvantages of PCR
**High sensitivity compared to culture and staining**	Potentially lower specificity compared to culture and staining
**Ability to test for anti-microbial resistance**	Need for narrow list of causative agents to use specific primers
**Quickly performed in 4-8 hours**	Possibility of amplifying normal flora from corneal scrapings
**Shown to be more cost-effective with selective use than culture and staining**	Becomes less cost-effective when performed with a multi-organism PCR approach
**Increased ability to detect less common organisms such as viruses**	Supply costs, machinery fees, training expenses

**Cost-Effectiveness**

Culturing and staining have been used for decades in large part due to their ease and accessibility. Staining and culturing are both inexpensive, with many of the materials widely available. In contrast, PCR requires trained technicians and specific machinery. Clinicians who wish to analyze PCR off-site can send samples to an outside laboratory, which can cost from 15 USD to 30 USD per microorganism tested [21]. The general approach to PCR commonly includes testing panels on a wide array of organisms using universal primers [22]. The cost per sample from this broad-range PCR approach can be hundreds of dollars [22]. The U.S. spends USD 175 million in total health expenditure annually on medical, non-surgical interventions of infectious keratitis [1, 4]. This value only includes costs associated with outpatient and emergency department visits that did not lead to subsequent hospital admissions, resulting in a significant underestimation of the expenditure [1]. Therefore, PCR must also be cost-effective to be widely used in the diagnosis of infectious keratitis. 

A cost-effective analysis comparing multiplex PCR to blood culturing of Candida infections showed a lower overall cost of PCR along with better health outcomes [17]. The estimated cost savings for the institution performing the analysis was 326,400 USD [17]. Other reports have suggested similar findings [23-25]. The cost to treat infectious keratitis significantly varies depending on the cause and severity of the infection [23]. By quick identification of the offending agent, PCR can lead to earlier treatment and reduction in vision loss, indirectly reducing the overall cost associated with infectious keratitis [23]. For example, PCR shows higher specificity for M. tuberculosis than staining, resulting in decreased costs by treating fewer misdiagnosed patients [24]. When used in conjunction with acid-fast stain to identify the causative agent, PCR is almost four times more cost-effective compared to a combination of stain and culture [25]. Infectious keratitis predominantly affects developing countries. Thirty times more people in India suffer from the disease each year compared to the United States [4, 5]. Even with the additional costs of performing PCR, these results suggest that a centralized lab performing PCR may be beneficial for countries disproportionately affected by infectious keratitis.

**PCR Use in Antibiotic Resistance**

Empiric therapy for infectious keratitis using broad-spectrum antimicrobials has led to antimicrobial resistance [4]. For example, in the case of Candidal keratitis, this type of clinical approach has led to antifungal resistance and increased expenditure [17]. The trend of antimicrobial resistance is especially evident in the case of bacterial keratitis. Cases of moxifloxacin resistance, a broad spectrum fluoroquinolone, has been documented in 26% of bacterial keratitis patients at a clinic in the United States [4]. In India, 60-66% of the methicillin-sensitive Staph aureus tested were resistant to moxifloxacin [26].

A benefit of PCR is the ability to test for multi-drug resistance. As shown with MRSA, a strength of multiplex PCR is the ability to test for multiple antibiotic genes with one sample, guiding specific antimicrobial treatment while reducing the chance of developing drug resistance [14]. Interestingly, another way PCR can evaluate sensitivity to antimicrobials is by quantitative analysis of viral load after treatment. Inoue et al. report a case of Herpes Simplex Virus that showed persistent levels of viral DNA on PCR after treatment with Acyclovir. This led physicians to prescribe trifluorothymidine [27].

## CONCLUSION

The future of PCR appears promising. New versions of the classic PCR have drastically shortened the time required to reach a diagnosis while also reducing the number of false positives [27-29]. Even in its present state, PCR has shown to match and even exceed the gold standard (culture and staining) in certain performance measures, such as sensitivity for the detection of microorganisms in infectious keratitis. The widely used broad-range PCR assay may distinguish among the various microbes. However, clinicians who wish to confirm clinical impression and consider cost-effectiveness may narrow the differential of causative organisms when sending samples to laboratories for PCR analysis. 

## DISCLOSURE

Ethical issues have been completely observed by the authors. All named authors meet the International Committee of Medical Journal Editors (ICMJE) criteria for authorship of this manuscript, take responsibility for the integrity of the work as a whole, and have given final approval for the version to be published. No conflict of interest has been presented.

## Funding/Support:

Research to Prevent Blindness, NY, USA
